# Secretion and activity of antimicrobial peptide cecropin D expressed in *Pichia pastoris*

**DOI:** 10.3892/etm.2012.719

**Published:** 2012-09-24

**Authors:** CHUNHE GUO, YUMAO HUANG, HONGYU ZHENG, LIYUN TANG, JUN HE, LINSHENG XIANG, DEHUI LIU, HOUQUAN JIANG

**Affiliations:** College of Veterinary Medicine, South China Agricultural University, Guangzhou, Guangdong 510642, P.R. China

**Keywords:** antimicrobial peptide cecropin D, *Pichia pastoris*, antibacterial activity

## Abstract

To express the antimicrobial peptide cecropin D in *Pichia pastoris* and determine the activity of the expressed product, four oligonucleotide fragments were synthesized in accordance with the available cecropin D sequences and a codon bias suitable for *Pichia pastoris*. Sequence fragments were phosphorylated, annealed, linked and cloned into the expression vector pGAPZαA and the yeast α-mating factor signal peptide was used as the signal sequence. The *P. pastoris* SMD1168 cells were transformed by electroporation using the constructed recombinant plasmid pGAPZαA-cecropin D. We were able to demonstrate by PCR that the cecropin D sequence had integrated into the *P. pastoris* genome. The expressed and secreted product was identified using Tricine-SDS-PAGE. Antibacterial activity was demonstrated using an agarose diffusion test and turbidimetry. The molecular mass of the recombinant cecropin D was estimated to be 3,900 Da. The recombinant cecropin D exhibited antibacterial activity for both Gram-positive and Gram-negative bacteria, suggesting that cecropin D was successfully expressed in *P. pastoris*. This approach holds great promise for antibacterial drug development.

## Introduction

Antimicrobial peptides (AMPs) are peptides which are generated by the innate immune system and confer specific immunity against microbial invaders. AMPs are able to effectively prevent pathogen invasion and possess broad-spectrum antibacterial activity against Gram-positive bacteria, Gram-negative bacteria, fungi, parasites, viruses and tumor cells. One of their most notable features is that AMPs rarely induce bacterial resistance which is a serious problem with conventional antibiotics (1). Therefore, AMPs have emerged as one of the most promising candidates for a new class of antibiotics.

Cecropins are small molecules with molecular sizes ranging from 3,500 to 4,000 Da and are considered to be the most potent AMPs. Structurally, cecropins possess a strong basic amino (N)-terminus and a long hydrophobic carboxyl (C)-terminus interrupted by a hinge region composed of a Gly-Pro sequence (2). Cecropins were first isolated from *Hyalophora cecropia* (3,4). Since then members of the cecropin-family peptides have been purified from *Manduca sexta* (5), *Bombyx mori* (6), *Drosophila melanogaster* (7) and *Sarcophage peregrine* (8). Cecropin D was chemically synthesized and its antibacterial activity was analyzed to allow the development of peptide antibiotics based on the structure of cecropin (9).

*Pichia pastoris* serves as a eukaryotic expression system and has been used successfully to produce recombinant heterologous proteins of human, animal, plant, fungal, bacterial and viral origins (10). The expression of secreted recombinant proteins in *P. pastoris* offers several advantages over bacterial expression systems. These include appropriate folding of molecules and disulfide bond formation, as well as execution of post-translational modifications which conserve protein function. Secretion of recombinant proteins circumvents intracellular accumulation, a significant aspect in the expression of toxic proteins. The secretion of recombinant proteins simplifies their purification by avoiding contamination with intracellular proteins (11,12). These advantages make secreted recombinant protein production in *P. pastoris* popular for scientific research. For this study, the SMD1168 strain was selected since it produces low levels of proteases. The present study demonstrates the successful manipulation of cecropin D for expression in yeast and the secretion of a functional AMP.

## Materials and methods

### 

#### Strains and plasmid

*P. pastoris* strain SMD1168 and expression vector pGAPZαA were purchased from Invitrogen (Carlsbad, CA, USA). The *Escherichia coli* strain DH5α and *Staphylococcus aureus* strain Cowan I were stored at the College of Veterinary Medicine, South China Agricultural University (Guangzhou, Guangdong, China).

#### Reagents and materials

rTaq DNA polymerase, DNA marker, T4 DNA ligase and the restriction enzymes *Xho*I, *Xba*I and *Bln*I were purchased from Takara Bio, Inc. (Shiga, Japan). Zeocin™ was obtained from Invitrogen. The E.Z.N.A Gel Extraction kit, Plasmid Mini kit and Cycle-Pure kit were purchased from Promega (Madison, WI, USA). The routine reagents were domestic and analytically pure.

#### Design of primers and synthesis of cecropin D

The cecropin D gene sequence from GenBank (serial No. NM_001043368.1) and the optimal codon list for *P. pastoris* were employed to design four primers using the Primer Premier 5.0 software (www.PremierBiosoft.com). The region amplified by the four primers covered the complete cecropin D coding region. The length of the amplicon was 108 bp. The four primer sequences were as follows: P1: 5′-CCATTCAAG GAGTTGGAGAGAG CTGGTCAAAGAGTCAGAGACGCTATCATCTCCGCT-3′; P2: 5′-CAAAGCGGTAGCTTGAGCGACGGTAGCGACA GCTGGACCAGCGGAGATGATAGCGTC-3′; P3: 5′-CCCT CGAG**AAAAGA**TGGAACCCATTCAAGGAGTTGGAG-3′ (containing an *Xho*I site, underlined; KEX2 signal cleavage site, bold); P4: 5′-GCTCTAGATTAGTTCTTAGCCAAAGC GGTAGCTTGAGC-3′ (containing an *Xba*I site, underlined). The sequence encoding cecropin D and the proteolytic signal KEX2 fused to cecropin D was synthesized by PCR using synthetic oligonucleotides.

The splicing by overlap extension (SOEing) PCR method was employed to synthesize the cecropin D gene sequence. The reaction mix for the PCR contained 5 *μ*l 10X PCR Buffer (Mg^2+^), 4 *μ*l dNTPs (2.5 mmol/l), 1 *μ*l each of the primers P1, P2, P3 and P4 (40 pmol/l), 0.25 *μ*l r*Taq* DNA polymerase and 34.75 *μ*l distilled water. Reactions were performed on a GeneAmp PCR System 2400 (Applied Biosystems, Carlsbad, CA, USA). PCR conditions were as follows: 4 min denaturation at 94°C, then 30 cycles (94°C for 30 sec, 65°C for 45 sec and 72°C for 30 sec) followed by a final 10 min elongation step at 72°C. The E.Z.N.A Gel Extraction kit was used to purify the PCR product. Samples (2 *μ*l) were loaded onto a 1% agarose gel and electrophoresed at 100 V for 15 min. The gel was scanned on a FluorImager SI (Molecular Dynamics, La Jolla, CA, USA).

The obtained 108-bp fragment was cloned in frame with the α-mating factor signal peptide of *S. cerevisiae* contained in the expression pGAPZαA vector. The insertion of this DNA fragment into the pGAPZαA vector replaced the original C-terminal proteolytic recognition sequences (containing a KEX2 and a STE3 site) with a sequence that contained only the KEX2 protease site.

#### Cloning of cecropin D into the yeast expression vector pGAPZαA and transformation of P. pastoris SMD1168

The PCR product was digested with the *Xba*l and *Xho*l restriction enzymes and ligated into the linearized pGAPZαA (Invitrogen) digested with the same enzymes. Following purification with an E.Z.N.A Cycle-Pure kit, the ligation product was transformed into competent *E. coli* DH5α cells. Restriction endonuclease analysis, PCR and sequencing were performed to validate the reading frame of the recombinant plasmid pGAPZαA-cecropin D.

*P. pastoris* was transformed by electroporation. Firstly, 5–10 *µ*g of the recombinant plasmid pGAPZαA-cecropin D was digested with *Bln*I and a small aliquot of the digest was used to confirm complete linearization by agarose gel electrophoresis. Competent yeast cells (80 *μ*l) were mixed with 5–10 *μ*g of linearized DNA (in 5–10 *μ*l sterile distilled water). The cell mixture was transferred to an ice-cold 0.2 cm electroporation cuvette and kept on ice for 5 min, then pulsed at 305 V for 15 msec. Ice-cold 1 M sorbitol (1 ml) was added to the cuvette immediately following electroporation. Aliquots of 10, 25, 50, 100 and 200 *μ*l were spread on separate yeast extract peptone dextrose (YPD) sorbitol plates containing 100 *μ*g/ml Zeocin. Plates were incubated for 2 to 3 days at 30°C until colonies had formed. Finally, 6 to 10 Zeocin-resistant *P. pichia* transformants were analyzed for the presence of an insert using PCR, or for copy number using Southern blot analysis.

#### Expression of cecropin D in P. pastoris SMD1168

A single colony was inoculated into 10 ml of YPD and grown at 28–30°C in an agitating incubator (250–300 rpm) overnight. An aliquot of 0.1 ml of this overnight culture was used to inoculate 50 ml of YPD in a 250-ml flask which was grown at 28–30°C in an agitating incubator (250–300 rpm). At the time points indicated below, 1 ml of the expression culture was transferred into a 1.5-ml microcentrifuge tube. These samples were used to analyze expression levels and to determine the optimal time to harvest the samples. Cultures were centrifuged at maximum speed in a tabletop microcentrifuge for 2–3 min at room temperature. Supernatants were transferred into a separate tube and analyzed by Tricine-SDS-PAGE. Samples were taken at the following time points: 0, 24, 48, 72 and 96 h.

#### Determination of the protein concentration

The Bradford Protein Assay kit was used to determine the protein concentration. Bovine serum albumin (BSA) was used as standard protein (0.5 mg/ml) and diluted with PBS in a 96-well plate. Cecropin D supernatant was diluted 1:1 with PBS and 200 *μ*l G250 staining solution was added to each well. A microplate reader was used to determine A_570_ which was used to estimate the total protein in the supernatant and controls. A light density scanner was used to scan the SDS-PAGE gel to assess the percentage of the target band in relation to total protein. These data were used to calculate the expressed level of the AMP cecropin D.

#### Antimicrobial assay

The antibacterial activity of cecropin D was tested using several Gram-positive and Gram-negative bacteria (listed in Table I) as described by Minagawa *et al* (13). A plate containing 1% agarose in 50 mM phosphate buffer (pH 6.2) and 10 *μ*l Gram-positive or Gram-negative bacteria was prepared. After solidification, four wells (2 mm in diameter) were carved into the plate; 30 *µ*l of the supernatant of SMD1168-pGAPZαA-cecropin D was loaded into one of the wells, while an equal amount of the supernatant of SMD1168-pGAPZαA and SMD1168 (negative controls) were loaded into two other wells. Ampicillin (2 *μ*l, 100 mg/ml) was loaded into the remaining well as a positive control. The plate was incubated at 37°C for 6–8 h and the diameters of the cleared zones were measured.

The minimal growth inhibition concentration (MIC) was determined using a liquid growth inhibition assay (14), with MIC defined as the lowest final concentration of the peptide at which no growth was observed (15,16). A stock solution of cecropin D was serially diluted 10-fold with 0.01% acetic acid and 0.2% BSA. Aliquots of each dilution were distributed into a 96-well polypropylene microtiter plate and each well was inoculated with a 100 *μ*l suspension of mid-log phase bacteria in LB broth. Cultures were grown for 24 h with vigorous agitation at 30°C and bacterial growth was evaluated by measuring the absorbance of the bacterial culture at 600 nm using a spectrophotometer.

### Tolerance of cecropin D to adverse conditions (high temperature, acid/base exposure, proteases)

#### High temperature tolerance

Supernatant from the expression culture of cecropin D was boiled for 15, 30 and 45 min. The antibacterial activity of the heat-treated supernatants was measured at the same time and under the same conditions as untreated supernatants which served as a control.

#### Acid and base tolerance

Cecropin D-containing super-natant was treated with HCl or NaOH (1 M) for 15, 30 and 45 min, after which time the same volume of NaOH or HCl (1 M) was added in order to neutralize the solution while using a pH meter. The antibacterial activity of the treated supernatants was detected as described previously using untreated control supernatants in parallel.

#### Tolerance to proteases

Supernatant from the expression culture of cecropin D was treated with trypsin and pepsin for 15, 30 and 45 min in a water bath at 37°C. Antibacterial activity in the supernatant was detected using an agarose diffusion test in the presence of appropriate controls.

#### Optimization of fermentation conditions

In order to improve the expression levels of cecropin D, the carbon source glucose was compared with glycerol under identical fermentation conditions. Urea was compared with tryptone and ammonium sulfate as a nitrogen source. In fed batch culture studies, carbon and nitrogen sources were supplied intermittently and compared with a regular supply of the same source.

## Results

### 

#### Cecropin D characterization and expression analysis

The sequence of the cloned cecropin D was compared with the sequence obtained from GenBank (Table II). The base composition was mostly identical and differed only in one base at position 40 where a change from Ala to Val had occurred.

**Figure 1:**
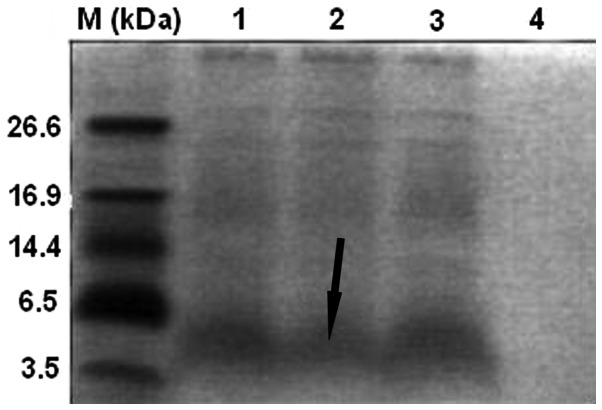
Tricine-SDS-PAGE of *P. pastoris* culture supernatants. M, low molecular weight protein marker; lanes 1–3; SMD1168 containing pGAPZαA-cecropin D; lane 4, SMD1168 containing pGAPZαA. The arrow indicates the band 3,900 Da of *P. pastoris* culture supernatant of SMD1168 containing pGAPZαA-cecropin D.

A major band of the polypeptide of interest (3.9 kDa; Fig. 1) was observed by Tricine-SDS-PAGE in the supernatant of cultures after 3 days of expression under optimal conditions. This band was absent from the control cultures. This result suggested that the polypeptide was successfully expressed in yeast. Secretion of the molecule into the supernatant further suggested that the signal peptide had been removed from the N-terminus of cecropin D.

**Figure 2:**
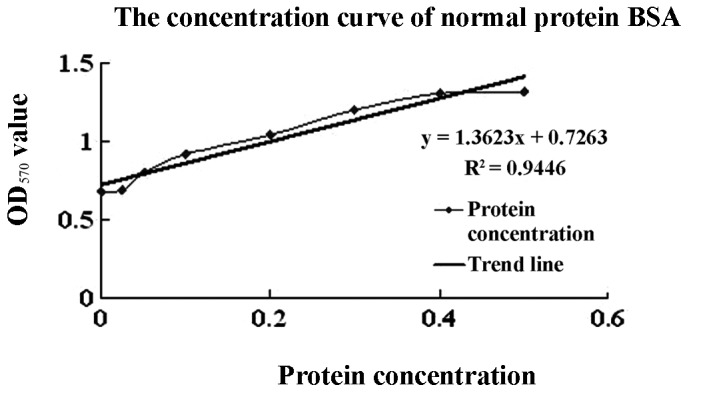
Protein standard curve of BSA.

A protein standard concentration curve was generated using various concentrations of BSA (Fig. 2). The OD_570_ of samples expressing cecropin D was 1.0506 and the percentage of the target protein was 85.34% of total protein determined by the light density scanner method. Using this information, expression levels of recombinant cecropin D were calculated to be 485.24 mg/l.

#### Antibacterial activity of recombinant cecropin D and MIC

The antimicrobial activity of cecropin D was tested against Gram-positive and Gram-negative bacteria. The diameters of the cleared zones were measured and the values are shown in Table I. The average diameter of the cleared zones ranged from 16–22 mm for the various bacterial species included in the experiment, while the cleared zones were absent from the negative controls. These data suggest that cecropin D exhibited antibacterial activity to Gram-positive and Gram-negative bacteria.

#### Tolerance of recombinant cecropin D to adverse conditions

Heat treatment of culture supernatants containing cecropin D had no effect on the antibacterial activity of cecropin D suggesting that cecropin D is insensitive to high temperatures.

Similarly, treatment of cecropin D using HCl or NaOH for 15 or 30 min had no influence on its antibacterial activity. The clear zones observed in bacteria lawns were similar in diameter to the samples that were not treated. However, when cecropin D was treated for 45 min with either HCl or NaOH, the diameters of the cleared zones decreased to 4 mm for *E. coli* K99, 5 mm for *Streptococcus equi ssp. zooepidemicus (SEZ)*, 3 mm for *B. pumilus* and 5 mm for *S. aureus* strain Cowan I, but not for any of the other bacteria species included.

Cecropin D in culture supernatant was exposed to trypsin and pepsin for 15, 30 and 45 min. There was no difference in the diameter of the cleared zones in the antibacterial assay between protease-treated supernatants and controls. This indicated that proteases did not have any effect on the antibacterial activity of cecropin D.

#### Optimization of fermentation

**Figure 3:**
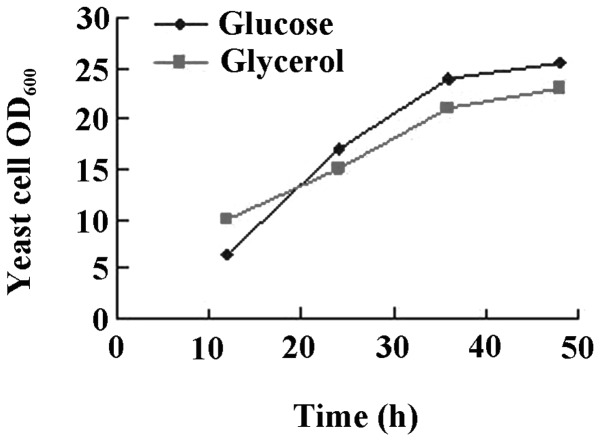
Growth of *P. pastoris* using glucose and glycerol as carbon source

**Figure 4:**
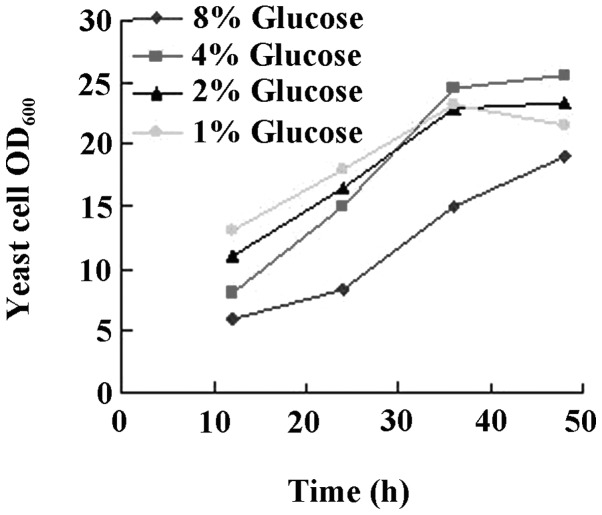
Growth of *P. pastoris* using various concentrations of glucose.

**Figure 5:**
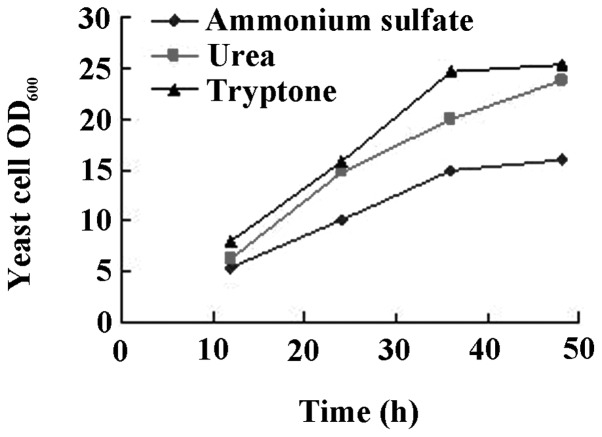
Growth of *P. pastoris* using various nitrogen sources (urea, tryptone and ammonium sulfate).

**Figure 6:**
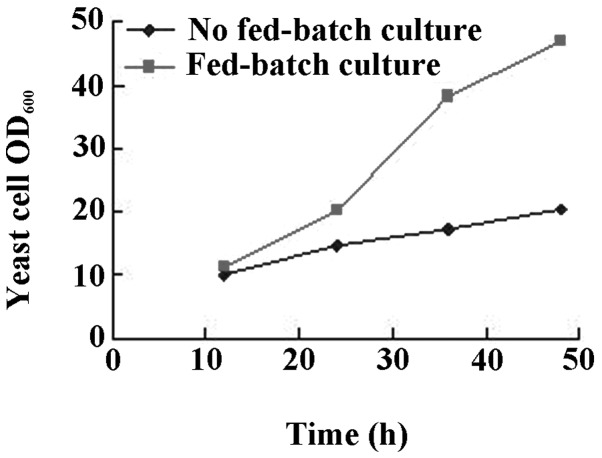
Fed batch cultures of *P. pastoris*.

Initial experiments were performed to explore whether it was possible to improve the expression level of cecropin D when using various carbon or nitrogen sources and with various initial concentrations of glucose. Under the assumption that improved yeast cell growth would lead to increased expression levels of the peptide, the change in OD_600_ of the yeast cells per 12 h was determined as an indicator of yeast cell growth. The results are shown in Figs. 3–6. While there was no difference between glucose or glycerol as the carbon source (Fig. 3), initiation of the cultures with 4% glucose had a beneficial effect on yeast growth (Fig. 4). When urea was used as the nitrogen source, the level of cell growth was improved compared with ammonium sulfate. However, there was no significant difference between urea or tryptone as nitrogen source (Fig. 5). Intermittent batch feeding had a beneficial effect on cell production as shown in Fig. 6.

## Discussion

In the present study the cloning of the cecropin D gene and its successful expression in *P. pastoris* were reported. A base mutation that was found in the cloned cecropin D gene may have occurred as an error in PCR, sequencing or may be associated with changes to the codon bias for *P. pastoris*. However, optimizing the codons of the gene to the preferred codon usage of *P. pastoris* was required to increase expression to the desired levels. To improve the expression levels of cecropin D, the protease-deficient *P. pastoris* strain SMD1168 was selected, high-copy transformants at a high concentration of Zeocin were screened for and appropriate concentrations of EDTA formaldehyde, or protease inhibitors were added to prevent protein degradation. To optimize the conditions for large-scale fermentation, initial experiments were performed to explore various carbon and nitrogen sources and their effects on fermentation conditions. Considering cost and expressed peptide levels, using glucose as the carbon source was superior to glycerol, while urea as a nitrogen source was superior to tryptone or ammonium sulfate. In addition, the intermittent addition of carbon and nitrogen (fed-batch culture) was more conducive to the growth of yeast cells. The amount of dissolved oxygen in the fermentation tank was vital to the growth of yeast cells and had a positive effect on the expression levels. Particularly on the third day of fermentation, a higher dissolved oxygen was required to guarantee a pH between 5.5 and 6.0 in the fermentation broth.

The *P. pastoris* expression system has been developed into an excellent tool for the large-scale expression of proteins from various sources (17). Its advantages are the ability to perform many of the post-translational modifications of higher eukaryotes and to secrete high levels of heterologous proteins into the supernatant under the control of the glyceraldehyde-3-phosphate dehydrogenase (GAP) promoter. *P. pastoris* also differs from bacterial systems in that the vector containing the desired gene is integrated into the genome during transformation (homologous recombination) (18). Furthermore, the vector pGAPZαA does not require methanol for induction, high levels of which are toxic to cells (19), making pGAPZαA an extremely convenient expression vector. In the present study cecropin D was demonstrated to be functionally expressed in *P. pastoris*. However, *P. pastoris* secretes various native proteins and these must be removed during the purification process of the desired recombinant protein. To enhance the utility of *P. pastoris*, a detailed profile of host-secreted proteins with regard to their identities and physical properties may provide critical insights for improving recombinant protein secretion and purification. D-glucose concentration is a key parameter affecting protein expression levels in the *P. pastoris* expression system. While the expression levels of cecropin D in *P. pastoris* were increased with D-glucose concentration, overly high D-glucose concentrations were unfavorable for protein expression. This result was similar to that obtained by Johnson *et al* (20).

It has been reported that the native N-terminal segment is a prerequisite for maintaining the activity of antibacterial peptides (21). Previously, cecropin1 from *M. domestica* was expressed in bacterial cells as a fusion protein with glutathione S-transferase. Recombinant cecropin1 was obtained following thrombin digestion but additional amino acid residues at the N-terminus of the recombinant cecropin1 decreased its activity against microbes (22). *Pisum sativum* defensin 1 (Psd1) was expressed in *P. pastoris*, but the recombinant Psd1 contained 4 additional amino acids (EAEA) at the N-terminus. In comparison with native Psd1, the anti-fungal activity of the recombinant Psd1 was decreased by at least 10-fold (23). In order to obtain cecropin D with the native N-terminus, the gene encoding cecropin D including the KEX2 cleavage site was cloned. Tricine-SDS-PAGE revealed that cecropin D was successfully secreted into the culture supernatant using the α-mating factor signal sequence. Activity assays demonstrated that cecropin D had a low MIC against Gram-negative and Gram-positive bacteria. Therefore, the α-factor signal sequence was efficient at secreting recombinant proteins into the culture medium and the signal peptide was efficiently processed by the KEX2 protease of *P. pastoris*.

The large-scale production of peptide antibiotics such as cecropin provides a significant challenge for developing commercial products. Bacterial expression systems may not be used to express cecropin and cecropin-like peptides, such as sarcotoxin IA, directly (24). Only small amounts of sarcotoxin IA were obtained when it was expressed in *Bombyx mori* cells using a baculovirus expression system (20 *μ*g/450 ml) (25) or in the yeast *Saccharomyces cerevisiae* (80 *μ*g/1) (26). In the present study, *P. pastoris* was used for the expression of cecropin D for the following reasons: firstly, it is fast and inexpensive to culture yeast and secondly, yeast cells are eukaryotes so they have the machinery for post-translational modifications (27).

In conclusion, the present study demonstrated that the antibacterial peptide cecropin D may be expressed in a heterologous expression system, *P. pastoris*, using the yeast secretion signal, α-mating factor. Recombinant cecropin D was expressed at high levels (up to 485.24 mg/l culture medium) and exhibited antimicrobial activity against Gram-positive and Gram-negative bacteria. Taken together, the results demonstrate that *P. pastoris* is a robust system for expressing the secreted form of recombinant cecropin D.

## Figures and Tables

**Table I:** Diameter of cleared zones of cecropin D exhibiting antibacterial activity towards Gram-positive and Gram-negative bacteria.^a^

	Microorganism						
	*E. coli* DH5α	*S. aureus* CowanI	*E. coli* K88	*Streptococcus* (SEZ)	*S. choleraesuis*	*B. pumilus*	*E. coli* K99
Antimicrobial diameter (mm)							
No. 1	19	17	21	17	16	18	22
No. 2	21	16	20	18	18	15	23
No. 3	17	15	22	20	16	17	21
Average diameter	19	16	21	18.33	16.67	16.67	22
Standard deviation	1.63	0.82	0.82	1.25	0.94	1.25	0.82
MIC (*μ*g/ml)	2.17	4.55	2.05	3.13	4.62	2.12	1.98
Ampicillin (mm)	30	23	24	27	20	21	31

a The MIC and the positive control are shown in the bottom row. MIC, minimal inhibition concentration. SEZ, *Streptococcus equi ssp. zooepidemicus*.

**Table II:** Cecropin D gene sequences obtained from GenBank and the sequence cloned into expression vector pGAPZαA-cecropin D.

Source	Gene sequence
GenBank (ID):	TGGAACCCATTCAAGGAGTTGGAGAGAGCTGGTCAAAGA**GCC(Ala)**AGAGACGCT
	ATCATCTCCGCTGGTCCAGCTGTCGCTACCGTCGCTCAAGCTACCGCTTTGGCTAAG
Cecropin D Gene:	TGGAACCCATTCAAGGAGTTGGAGAGAGCTGGTCAAAGA**GTC(Val)**AGAGACGCT
	ATCATCTCCGCTGGTCCAGCTGTCGCTACCGTCGCTCAAGCTACCGCTTTGGCTAAG

Bold text highlights a difference in the gene sequence and the corresponding change in amino acid.
